# Rigorous Randomized Controlled Trial Implementation in the Era of COVID-19

**DOI:** 10.4269/ajtmh.20-0262

**Published:** 2020-04-15

**Authors:** Catherine E. Oldenburg, Thuy Doan

**Affiliations:** 1 Francis I. Proctor Foundation, University of California, San Francisco, San Francisco, California;; 2 Department of Ophthalmology, University of California, San Francisco, San Francisco, California;; 3 Department of Epidemiology and Biostatistics, University of California, San Francisco, San Francisco, California

A pneumonia of unknown cause was first reported to the WHO on December 31, 2019. In the following months, this disease rapidly evolved into a global pandemic and was named novel coronavirus disease (COVID-19) caused by the novel coronavirus severe acute respiratory syndrome coronavirus 2 (SARS-CoV-2). Although estimation of the case fatality rate remains challenging because of inconsistent testing and variable duration of illness, COVID-19 clearly causes substantial morbidity and mortality. Rapid identification of effective treatment and prevention strategies may be the most important thing we can do to prevent unnecessary loss of life. A rigorous approach to identifying treatment and prevention strategies is necessary, however, to avoid funneling resources into unproven or even potentially harmful therapies.

A major challenge in early response in a rapidly evolving epidemic caused by a novel pathogen is the lack of evidence on which to make treatment and prevention decisions. Early evidence of novel infections often comes from case series and other poorly controlled studies. In the era of rapid dissemination of information, this can easily translate into changes in standard of care or in public perceptions of what standard of care should be. For example, a non-randomized study purported to demonstrate a reduction in SARS-CoV-2 viral load after treatment with hydroxychloroquine plus azithromycin, and this report was tweeted more than 4,000 times in the 10-day period following its publication.^1^ On March 29, 2020, the Food and Drug Administration (FDA) issued an emergency use authorization to allow hydroxychloroquine sulfate and chloroquine phosphate to be used in hospitalized patients with COVID-19 when a clinical trial is not available. Although numerous placebo-controlled trials are ongoing or planned, the FDA announcement may paradoxically make it more difficult to implement and enroll in these trials, which may threaten our ability to generate an evidence base for treatment both during the current epidemic and in future coronavirus outbreaks.

Randomized placebo-controlled trials (RCTs) should remain the gold standard to guide clinical care, even in emergency settings. In the absence of a randomized control arm, we simply do not know the counterfactual. That a subset of patients recover following treatment is not evidence of efficacy when patients routinely clear infection without therapy. Non-randomized comparative studies are at high risk of bias due to confounding by indication, disease severity, and other factors. The use of hydroxychloroquine or other drugs under consideration for treatment of patients with SARS-CoV-2 does not come without risk, and eventual randomized evidence may prove disappointing. For example, despite case reports suggesting an effect of lopinavir–ritonavir against SARS-CoV-2,^2^ the first published randomized trial failed to find evidence of benefit in hospitalized patients with severe COVID-19.^3^

In a rapidly changing setting, traditional RCT rules may not apply. A traditional power calculation for an RCT is fixed, based on assumptions about known epidemiology of the disease of interest. With a novel emerging pathogen, the epidemiology is unknown. SARS-CoV-2 testing remains heterogeneous, and the true incidence of infection is unknown. In many areas, testing is currently mostly prioritized for the sickest patients, inflating the probability of outcomes such as hospitalization or mortality. As testing is made available more widely, this probability will likely decrease, which could lead to underpowered studies. Flexible or adaptive sample sizes, as have been proposed for studies during Ebola outbreaks,^4^ do not require a prespecified maximum sample size and allow for reassessment of parameters as the epidemic progresses.

Changes in standards of care or identification of new therapeutic candidates require flexibility in study arms. Community randomized trials of immediate antiretroviral therapy (ART) initiation for HIV had to accommodate changing country-level treatment guidelines, resulting in changes to the standard of care in control arms.^5^ Changing guidelines to earlier initiation of ART resulted in study arms with results more similar to each other than initially planned, requiring larger sample sizes to detect differences or result in futility. Furthermore, results of contemporaneous COVID-19 trials are likely to change the risk-benefit ratio of other randomized interventions. Candidate therapeutics may be identified during the course of ongoing trials, and an efficient approach to evaluating them may be incorporation into existing trials. Ensuring protocol flexibility to accommodate new evidence arising during the course of the epidemic will maximize the benefit of RCTs during the COVID-19 pandemic.

The existence of a pandemic does not lower the bar for standards of evidence. Under normal circumstances, trial implementation can take years. The pace of pandemics places additional pressure on studies to generate evidence quickly. The massive size of this pandemic and the short clinical course of COVID-19 facilitate rapid accrual of RCT data. Given the high stakes, the imperative for high-quality research is greater than ever. Flexible and reflective RCTs will likely improve the outcome of this pandemic and also provide road maps for the next pandemic.
